# Supplementary material for: Causal Relationship between Aging and Anorexia Nervosa: A White-Matter-Microstructure-Mediated Mendelian Randomization Analysis
Source: Biomedicines. 2024 Aug 16;12(8):1874. doi: 10.3390/biomedicines12081874 (PMC11351342; doi:10.3390/biomedicines12081874)
Supplement: Supplementary file 1 [file biomedicines-12-01874-s001.zip › Supplementary Figure/Figure S4/Singe SNP plot of MR analysis for TL to ubm-b-2057.pdf]

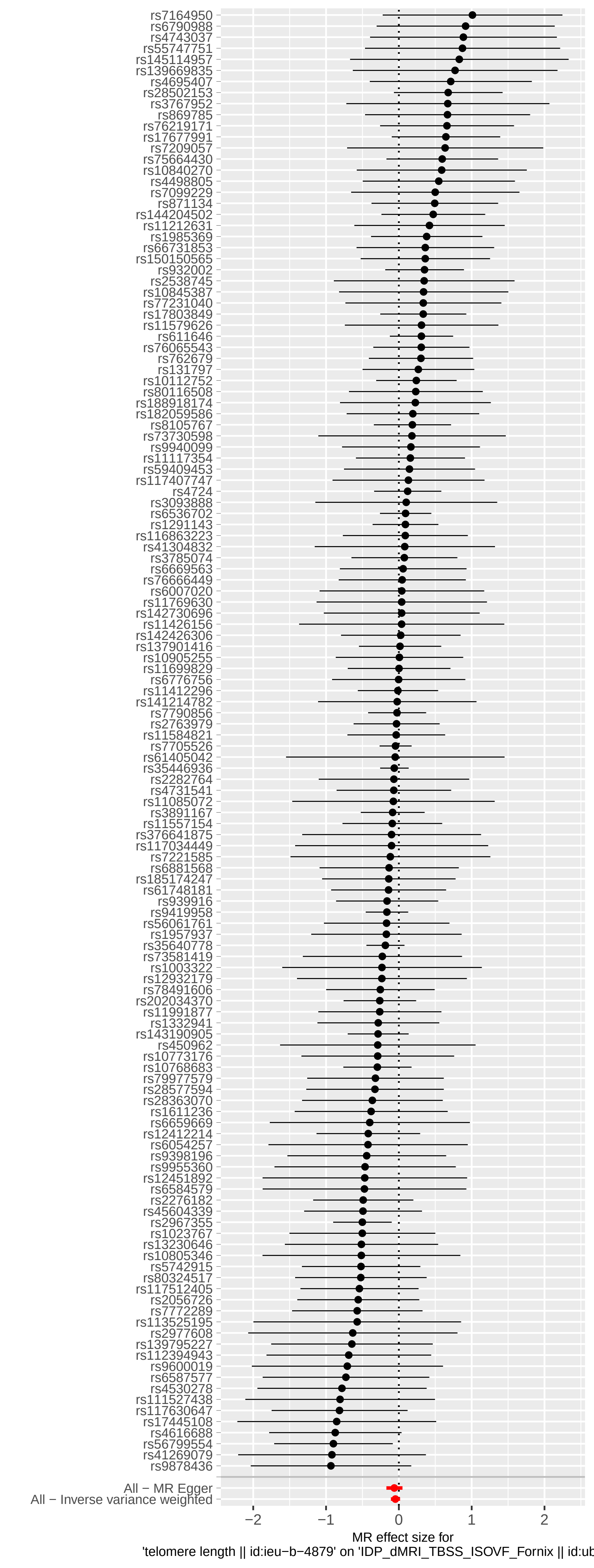
rs7164950
rs6790988
rs4743037
rs55747751
rs145114957
rs139669835
rs4695407
rs28502153
rs3767952
rs869785
rs76219171
rs17677991
rs7209057
rs75664430
rs10840270
rs4498805
rs7099229
rs871134
rs144204502
rs11212631
rs1985369
rs66731853
rs150150565
rs932002
rs2538745
rs10845387
rs77231040
rs17803849
rs11579626
rs611646
rs76065543
rs762679
rs131797
rs10112752
rs80116508
rs188918174
rs182059586
rs8105767
rs73730598
rs9940099
rs11117354
rs59409453
rs117407747
rs4724
rs3093888
rs6536702
rs1291143
rs116863223
rs41304832
rs3785074
rs6669563
rs76666449
rs6007020
rs11769630
rs142730696
rs11426156
rs142426306
rs137901416
rs10905255
rs11699829
rs6776756
rs11412296
rs141214782
rs7790856
rs2763979
rs11584821
rs7705526
rs61405042
rs35446936
rs2282764
rs4731541
rs11085072
rs3891167
rs11557154
rs376641875
rs117034449
rs7221585
rs6881568
rs185174247
rs61748181
rs939916
rs9419958
rs56061761
rs1957937
rs35640778
rs73581419
rs1003322
rs12932179
rs78491606
rs202034370
rs11991877
rs1332941
rs143190905
rs450962
rs10773176
rs10768683
rs79977579
rs28577594
rs28363070
rs1611236
rs6659669
rs12412214
rs6054257
rs9398196
rs9955360
rs12451892
rs6584579
rs2276182
rs45604339
rs2967355
rs1023767
rs13230646
rs10805346
rs5742915
rs80324517
rs117512405
rs2056726
rs7772289
rs113525195
rs2977608
rs139795227
rs112394943
rs9600019
rs6587577
rs4530278
rs111527438
rs117630647
rs17445108
rs4616688
rs56799554
rs41269079
rs9878436
All − MR Egger
All − Inverse variance weighted
−2
−1
0
1
2
MR effect size for
'telomere length || id:ieu−b−4879' on 'IDP_dMRI_TBSS_ISOVF_Fornix || id:ub
